# Painless, verrucous lesion of the penile shaft: An uncommon clinical case

**DOI:** 10.1016/j.jdcr.2025.06.050

**Published:** 2025-07-24

**Authors:** Lucy Wang, June Y. Moon, Kenneth Shulman, Banu Farabi

**Affiliations:** a Department of Dermatology, New York Medical College, Valhalla, New York; b Department of Dermatology, NYC Health + Hospital/Metropolitan, New York, New York; c Department of Dermatology, NYC Health + Hospital/South Brooklyn Health, Brooklyn, New York

**Keywords:** dermoscopy, herpes simplex virus, herpes vegetans, histopathology, HIV/AIDS, immunosuppression, infectious disease

## Case description

**Fig 1:**
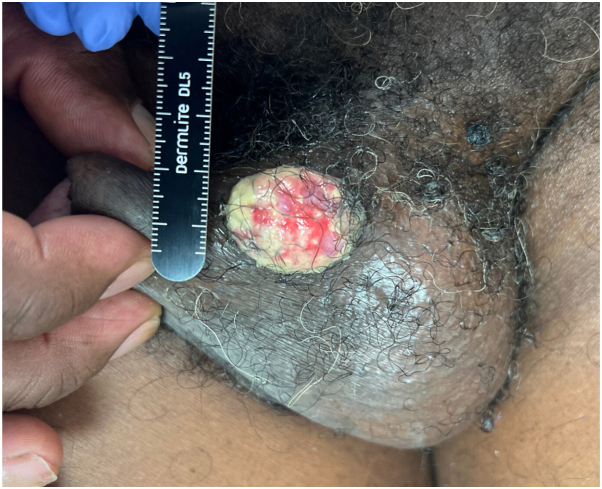
An exophytic, rapidly growing, painless mass measuring 4.2 × 3 cm at the base of his penile shaft.

**Fig 2:**
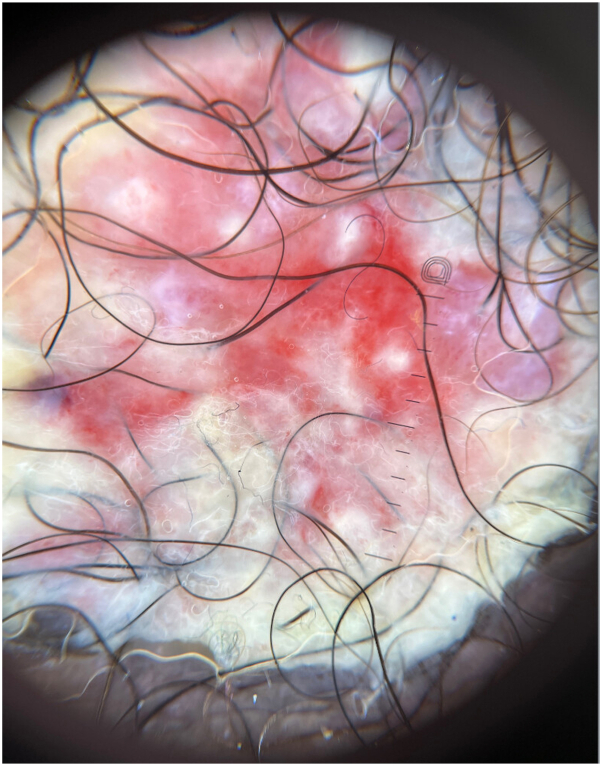
Dermoscopy showed a pink-to-red structureless background with scattered white areas and focal areas of erosion.

A 59-year-old male with well-controlled HIV, acyclovir-resistant herpes simplex virus (HSV), and chronic hepatitis B presented to the dermatology clinic with a 2-month-old, foul-smelling, rapidly growing, painless mass measuring 4.2 × 3 cm at the base of his penile shaft (Fig 1). Dermoscopy showed a pink-to-red structureless background with scattered white areas and focal areas of erosion (Fig 2). A biopsy of the lesion was obtained, and hematoxylin and eosin staining was performed on the lesion and its periphery. A recent visit to the emergency department revealed a nonreactive rapid plasma reagin and an absolute CD4 count of 407 cells/mm^3^.

**Question: What is the diagnosis?**

**A.** Mycobacterial infection
**B.** Condyloma lata
**C.** Herpes vegetans
**D.** Squamous cell carcinoma
**E.** Pyogenic gangrenosum

**Diagnosis: Herpes vegetans**

Herpes vegetans (HV) is a rare manifestation of HSV infection that primarily occurs in immunocompromised individuals, particularly those with HIV/AIDS, but also in individuals with cancer, undergoing chemotherapy or immunosuppressive therapy, or receiving organ transplants.^1^ HV can occur even in HIV-positive individuals with well-controlled disease, as demonstrated by our patient and prior reports.^2^ The proposed pathogenesis of HV reflects dysregulated host-virus interactions in immunocompromised states, where impaired T-cell-mediated immunity and diminished interferon-alpha responses permit disinhibited HSV replication, driving pseudoepitheliomatous hyperplasia and chronic inflammation.^3^

Unlike classic HSV infections, which present as grouped vesiculoulcerative lesions, HV develops as rapidly growing, ulcerative, exophytic, or verrucous masses with chronic progression,^4^ often mimicking neoplastic or granulomatous processes. HV lesions most commonly occur in the anogenital region, but cases have also been reported in the perioral and facial areas, shoulder, and buttocks, highlighting the HSV’s potential for disseminated infection in immunocompromised hosts.^1^

**Fig 3:**
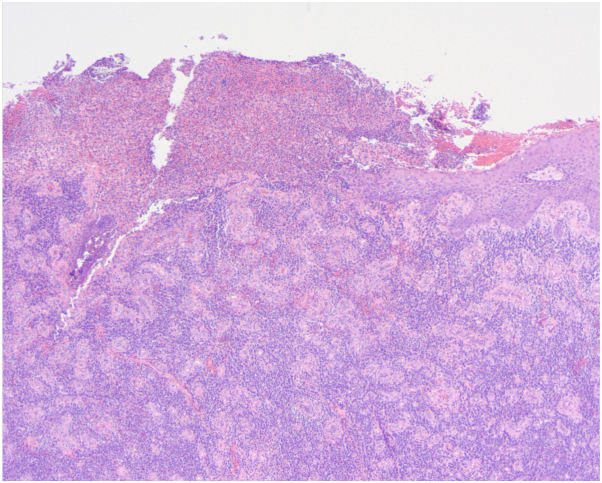
Hemotoxylin and eosin (H&E) staining, 4×.

**Fig 4:**
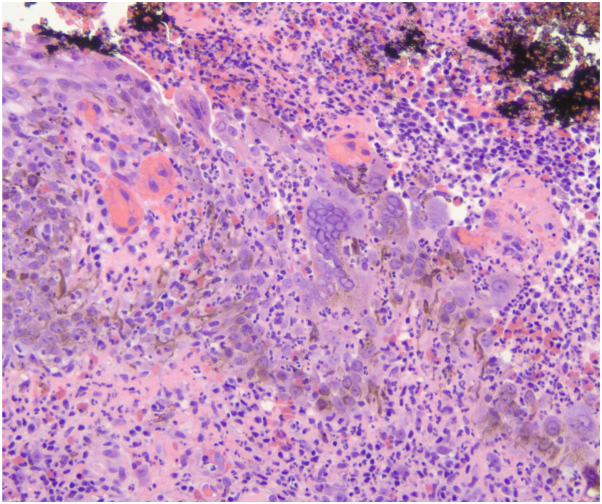
Hemotoxylin and eosin staining, 20×.

Histologic findings include epithelial hyperplasia, ulceration, chronic inflammatory infiltrate, and viral cytopathic changes such as eosinophilic intranuclear inclusions and multinucleated keratinocytes.^1^ Similarly, histopathologic findings from a biopsy of our patient revealed ulceration with loss of the epidermis and upper dermis associated with mixed infiltrate consisting of lymphocytes, eosinophils, neutrophils, and plasma cells (Fig 3), and viral inclusions in squamous epithelium with multinucleation, nuclear molding, and steel gray nuclei at the lesion periphery (Fig 4).

The hypertrophic morphology of HV results in a broad differential diagnosis that requires exclusion of various cutaneous conditions, including condyloma lata, squamous cell carcinoma, pyogenic gangrenosum, and fungal and mycobacterial infections. A high index of clinical suspicion for HV is crucial, especially in immunocompromised patients and even those with well-controlled HIV. Treatment of HV may involve acyclovir, first-line therapy for herpetic infections; however, acyclovir resistance is often present in HV, necessitating alternative agents such as foscarnet, cidofovir, or imiquimod.^5^

## Conflicts of interest

None disclosed.
